# Historical translocations by Māori may explain the distribution and genetic structure of a threatened surf clam in Aotearoa (New Zealand)

**DOI:** 10.1038/s41598-018-35564-4

**Published:** 2018-11-22

**Authors:** Philip M. Ross, Matthew A. Knox, Shade Smith, Huhana Smith, James Williams, Ian D. Hogg

**Affiliations:** 1 Environmental Research Institute, University of Waikato, Tauranga, New Zealand; 2 School of Science, University of Waikato, Hamilton, New Zealand; 3 Hopkirk Research Institute, Massey University, Palmerston North, New Zealand; 4 Triplefin Environmental Consultants, Napier, New Zealand; 5 Te Rangitāwhia Whakatupu Mātauranga Ltd, Kuku, New Zealand; 6 National Institute of Water and Atmospheric Research, Auckland, New Zealand; 7 Canadian High Arctic Research Station, Polar Knowledge Canada, Cambridge Bay, Canada

## Abstract

The population genetic structure of toheroa (*Paphies ventricosa*), an Aotearoa (New Zealand) endemic surf clam, was assessed to determine levels of inter-population connectivity and test hypotheses regarding life history, habitat distribution and connectivity in coastal vs. estuarine taxa. Ninety-eight toheroa from populations across the length of New Zealand were sequenced for the mitochondrial cytochrome *c* oxidase I gene with analyses suggesting a population genetic structure unique among New Zealand marine invertebrates. Toheroa genetic diversity was high in Te Ika-a Māui (the North Island of New Zealand) but completely lacking in the south of Te Waipounamu (the South Island), an indication of recent isolation. Changes in habitat availability, long distance dispersal events or translocation of toheroa to southern New Zealand by Māori could explain the observed geographic distribution of toheroa and their genetic diversity. Given that early-Māori and their ancestors, were adept at food cultivation and relocation, the toheroa translocation hypothesis is plausible and may explain the disjointed modern distribution of this species. Translocation would also explain the limited success in restoring what may in some cases be ecologically isolated populations located outside their natural distributions and preferred niches.

## Introduction

Dispersal and connectivity among populations of marine organisms are strongly influenced by a species’ life history characteristics^1^. Pelagic larval duration has been shown to be a reasonable indicator for dispersal potential^2–4^, which is further modified by spawning or larval behaviour and the physical properties of dispersing propagules^5–7^. Within the constraints imposed by a species’ biology, the physical environment also plays a role in determining the distances over which populations are connected. Local and regional hydrodynamics are important^8^, as is the geographic distribution of suitable habitat^9^. For example, estuarine taxa may exhibit greater genetic structure, indicative of limited connectivity, because they live in geographically discrete habitats^10,11^. In contrast, open-coast taxa are often better connected, through either larval or post-settlement dispersal, over similar geographic scales because their habitat is more continuous, allowing greater mixing among populations^12^. Consequently, estuarine species may be more vulnerable to overharvesting than coastal taxa as recruitment from distant estuaries may be infrequent or insufficient to restore or sustain impacted populations. A similar situation may exist for open-coast taxa with disjunct distributions. For species which occur under a relatively narrow range of environmental conditions, distances between populations may be great and inter-population dispersal rare^9,13^. One such open-coast organism that occurs in geographically discrete populations and is hypothesised to experience limited inter-population connectivity is the toheroa (*Paphies ventricosa*)^14^, a large intertidal surf clam endemic to Aotearoa (New Zealand).

Toheroa are broadcast spawners with a pelagic larval duration of around three to six weeks^15,16^. Toheroa inhabit exposed open-coast surf beaches and are primarily found in the middle of the intertidal zone. Juveniles are located at the upper end of this range (nearer the top of the beach) with adults located lower down the shore and buried up to 15–20 cm beneath the beach surface. The exact parameters that determine habitat suitability are uncertain (J. Cope *unpublished research*). However, in contrast to other New Zealand bivalves, such as the surf clam *Paphies subtriangulata* (tuatua) or the estuarine clam *Paphies australis* (pipi), toheroa appear to have habitat requirements that limit their geographic distribution^17^. Common features of the beaches on which toheroa occur include high wave energy conditions, a wide shallow gradient (dissipative beach) usually backed by sand dunes or cliffs, fine uniform sand with an average grain size of 0.21–0.33 mm, high levels of fresh water seepage onto the beach and high concentrations of phytoplankton^18,19^.

**Figure 1:**
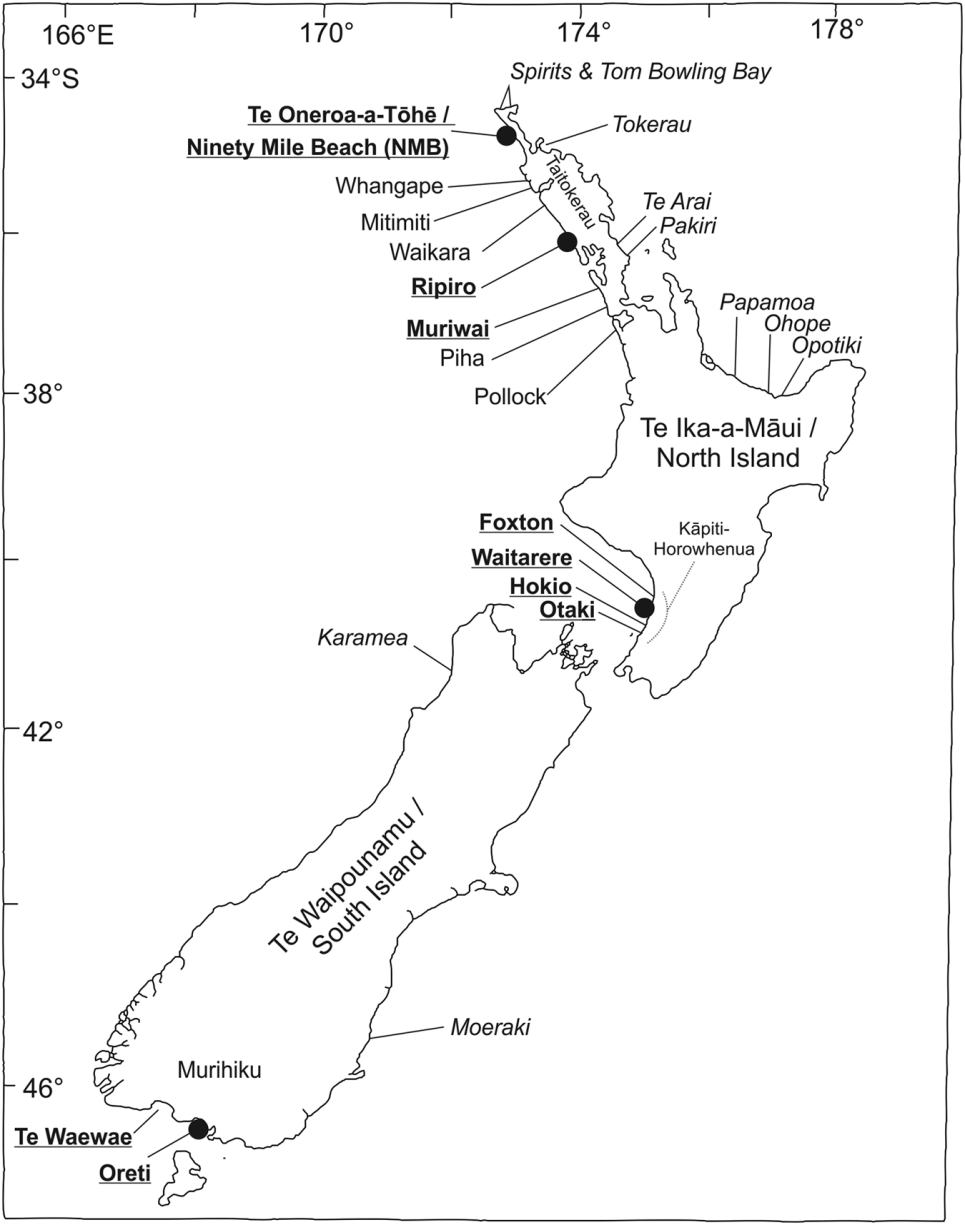
The distribution of toheroa (*Paphies ventricosa*) in Aotearoa (New Zealand). The locations where major populations have been recorded since the beginning of the 20^th^ century are indicated by bold and underlined text. Sites where toheroa have been anecdotally reported or where shells or small numbers of living toheroa have been occasionally reported are indicated in italics. Toheroa populations sampled for this study are indicated by black circles.

At the start of the 20th century, extensive toheroa populations were present on a handful of exposed west and south facing surf beaches (Fig. 1)^14^. Toheroa were a staple food for Māori (the indigenous people of New Zealand) in these areas and remain a species of great cultural importance. Toheroa began to be harvested more extensively by Pākehā (New Zealanders of European descent) from the late 1800s and intense harvesting over the next 60–70 years resulted in toheroa populations declining to levels where their harvest was no longer viable. Commercial and recreational fisheries were closed between 1969 and 1980^14^. Since that time only limited harvest for Māori customary purposes have been permitted. Despite having been protected for over 40 years, toheroa populations nationwide have, for unknown reasons, failed to recover, with some populations continuing to decline^14^.

The distribution of toheroa is unlike that of any other New Zealand marine invertebrate^2,14,17^ (Fig. 1). Toheroa are largely absent from New Zealand’s east coast and have a disjunct distribution over more than 1700 km (>11° latitude) of the western and southern coastline (Fig. 1). Notable populations have only been recorded in three regions. In Te Ika-a Māui (the North Island of New Zealand), toheroa can be found on the northland (Taitokerau) west coast from Muriwai to Te Oneroa-a-Tōhē (Ninety Mile Beach) and on the south-west (Kāpiti-Horowhenua) coast from Ōtaki to Foxton. In Te Waipounamu (the South Island) toheroa are largely restricted to Oreti Beach and Te Waewae Bay in Murihiku (the southern tip of the South Island) although there are accounts of small and transient populations in other southern locations. Numerous hypotheses have been proposed to explain the continued decline of toheroa including recruitment limitation and a lack of connectivity between geographically isolated populations^14^. Given the relatively short pelagic larval phase, and our knowledge of coastal circulation^20–22^, the exchange of toheroa larvae between northern and southern locations seems highly unlikely. Furthermore, a genetic boundary through central New Zealand is almost ubiquitous among marine invertebrates^2,23^, indicating possible barriers to north-south larval exchange.

In this study we assessed population genetic structure and levels of connectivity among toheroa across their entire distribution using the mitochondrial cytochrome *c* oxidase I (COI) gene. Our predictions, based on prior ecological knowledge, were for North Island populations to be well connected and for isolation and genetic divergence between northern and southern toheroa. Connectivity was expected to be less than that previously recorded for widely distributed open-coast taxa, such as *P. sutriangulata*, and more similar to levels of connectivity reported in studies of estuarine species such as *P. australis*^10^.

## Results

### Genetic diversity

**Figure 2:**
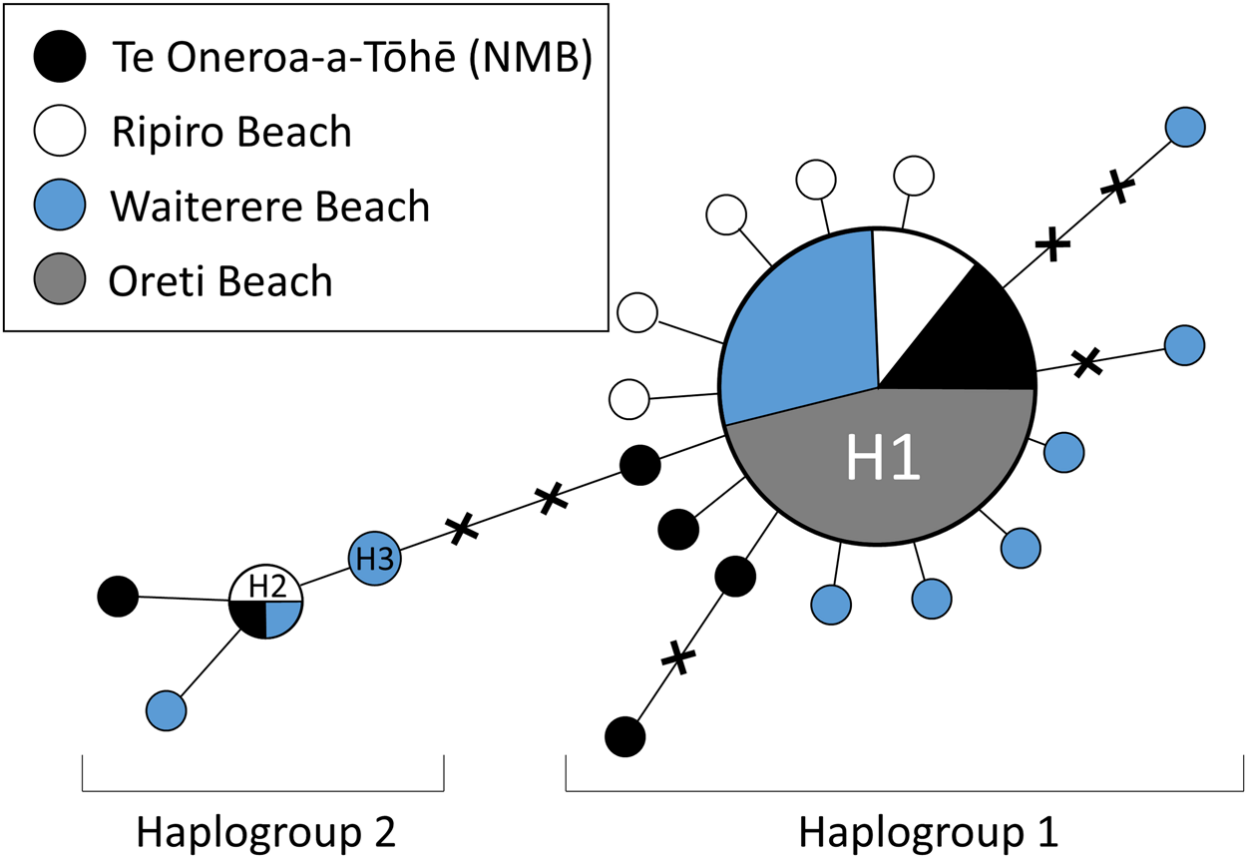
Haplotype Network of 98 toheroa (*Paphies ventricosa*) CO1 sequences. Each circle represents an individual haplotype with circle size indicating the number of individuals (*n*) sharing that haplotype (*n*(H1) = 75, *n*(H2) = 4 and *n*(H3) = 2). For all other haplotypes *n* = 1. Circle colour indicates the sampling location. Missing (unsampled) intermediate haplotypes are indicated by ‘**X’**s.

**Figure 3:**
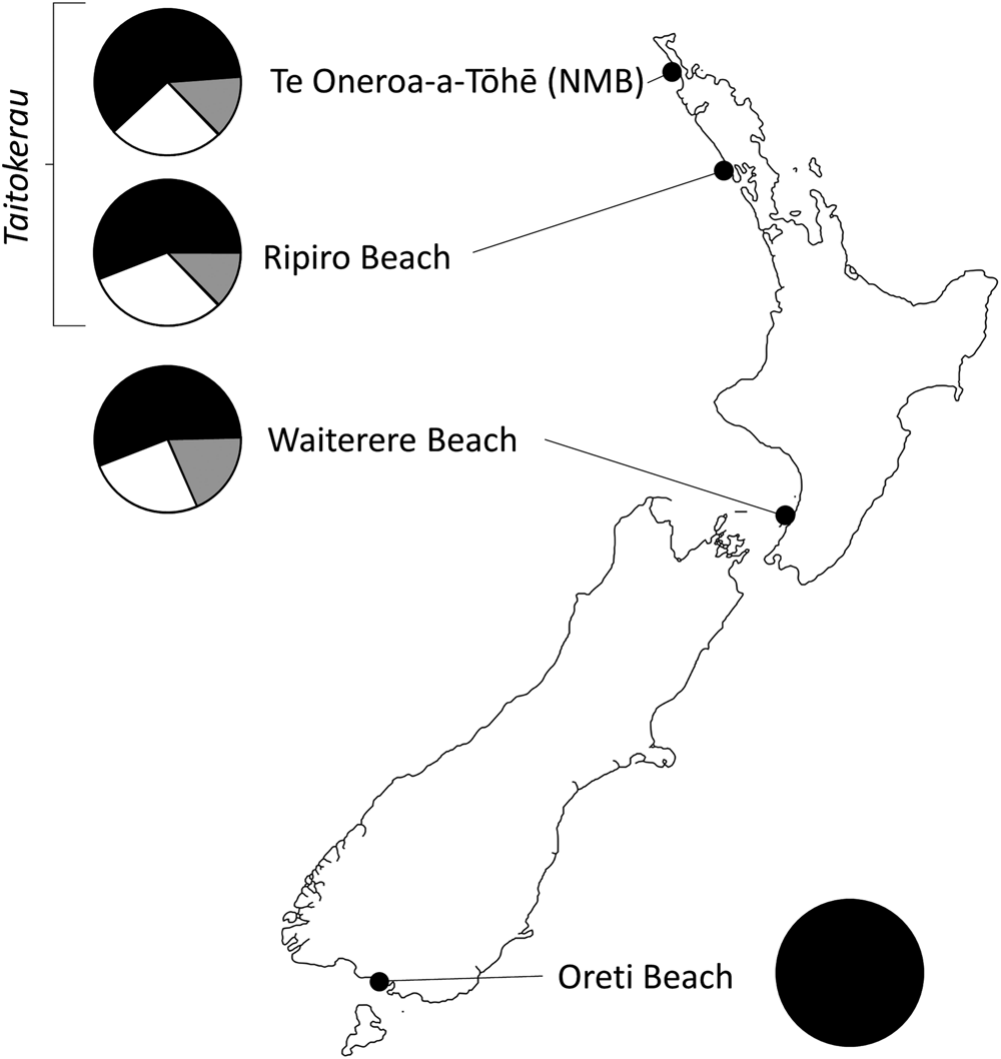
Map showing geographic distribution of toheroa (*Paphies ventricosa*) genetic diversity. Haplotype H1 is shown in black, other haplotypes from Haplogroup 1 (Fig. 2) are shown in white and haplotypes from Haplogroup 2 are shown in grey. Refer to Fig. 2 for haplotype network and haplogroups.

Toheroa from Te Oneroa-a-Tōhē (NMB, *n* = 16) and Ripiro Beach (*n* = 16) in Northland, Waitarere Beach (*n* = 30) on the Kāpiti-Horowhenua coast and Oreti Beach (*n* = 36) in Southland were sequenced generating a 485 nucleotide COI alignment. Twenty-three nucleotide positions were variable leading to the delineation of 20 haplotypes (Table 1). Haplotype and nucleotide diversity was comparable across North Island populations with 12 haplotypes recorded for NMB/Ripiro (hereafter referred to together as Taitokerau; H_D_ = 0.65, π = 0.0038) and 10 at Waitarere (H_D_ = 0.56, π = 0.0036). In contrast, only a single haplotype (H1) was recorded at Oreti (H_D_ = 0, π = 0). Haplotype H1 was also the most abundant haplotype across North Island populations, accounting for 59% and 66% of sequences from Taitokerau and Waitarere (Figs 2 and 3); Table 1). Of the remaining 19 haplotypes only one was shared among populations. Haplotype H2 was recorded at NMB (*n* = 1), Ripiro (*n* = 2) and Waitarere (*n* = 1). The remaining 18 haplotypes were private (unique to one location) with all but one of these recorded in just one specimen. A rarefaction curve generated from haplotype frequencies suggested that greater sampling effort could yield many more haplotypes, a notion supported by the number of missing intermediate positions indicated in a haplotype network (Fig. 2). Two groups of haplotypes were evident in this network (Fig. 2). Group 1, a star shaped haplogroup, contained 13 haplotypes separated from H1 by a single mutation and another three singleton haplotypes separated from H1 by one or two missing haplotypes. A second star shaped haplogroup was separated from the H1 haplotype by four to six mutations. This second group consisted of H2 and three additional haplotypes private to NMB and Waitarere.

**Table 1:** Summary statistics for toheroa (*Paphies ventricosa*) populations including number of COI sequences obtained (n), number of polymorphic sites (S), numbers of transitions (T_S_) and transversions (T_V_), numbers of haplotypes detected (H_N_), number of private haplotypes per location (H_P_), haplotype diversity (H_D_), nucleotide diversity (π), mean number of pairwise differences (k) and Tajima’s *D* and Fu’s Fs (with *p*-values).

Location	n	S	T_S_	T_V_	H_N_	H_P_	H_D_ (s.d.)	π (s.d.)	k	Tajima’s D	p	Fu’s Fs	p
Taitokerau	32	14	14	0	12	10	0.649 (0.096)	0.00380 (0.00095)	1.843				
*Te Oneroa-a-Tōhē (NMB)*	16	9	9	0	7	5	0.625 (0.139)	0.00404 (0.00140)	1.958	−1.02093	0.174	−1.780	0.097
*Ripiro*	16	10	10	0	7	5	0.629 (0.124)	0.00369 (0.00126)	1.792	−1.51227	0.053	−2.058	0.073
Waitarere	30	14	14	0	10	8	0.561 (0.109)	0.00359 (0.00098)	1.743	−1.69162	0.035	−3.815	0.017
Oreti	36	0	0	0	1	0	0	0	0	—	—	—	—
All locations	98	23	23	0	20	18	0.415 (0.064)	0.00240 (0.00051)	1.162	−2.18704	<0.001	−17.639	<0.001

Values are presented both individually for Te Oneroa-a-Tōhē and Ripiro and with these two collection sites combined (Taitokerau).

### Population genetic structure

AMOVA revealed statistically significant pairwise differences between all populations (p < 0.001; F_ST_ = 0.39–0.74; Table 2) with differentiation between Oreti and Taitokerau toheroa (NMB and Ripiro) being greatest (F_ST_ = 0.69–0.74; Table 2). Tajima’s D and Fu’s Fs were significantly negative for Waitarere (D = −1.692, p = 0.035; F = −3.815, p = 0.017) and for the COI data set as a whole (D = −2.187, p < 0.001; F = −17.639, p < 0.001; Table 1). This indicates evolution under non-random processes which could include directional selection and expansion or contraction. D and Fs could not be scored for Murihiku on account of the total lack of genetic diversity recorded at this site.

**Table 2:** F_ST_ values among all toheroa (*Paphies ventricosa*) populations (below diagonal) and significance (above diagonal).Sampling locations referred to are displayed in Fig. 1.

	Taitokerau	Waitarere	Oreti
Taitokerau	—	***	***
Waitarere	**0.394**	—	***
Oreti	**0.689**	**0.74**	—

Significant F_ST_ values are indicated in bold. ***p < 0.001.

## Discussion

This study provides support for the hypothesis that southern toheroa are isolated from toheroa in the North Island. However, the lack of genetic divergence between northern and southern populations is inconsistent with their distribution and dispersal capacity. Our results are also inconsistent with the expectation of connectivity among North Island toheroa, potentially indicating a dispersal capacity that is less than would be expected based on toheroa life history. The suggestion of expanding or contracting populations is consistent with toheroa dynamics over the last century as survey data indicate large fluctuations in toheroa abundance from year to year^24,25^. This dynamic was particularly evident in the decades leading up to the collapse and closure of the fishery, and is also relatively common in surf clams worldwide on account of their highly variable rates of recruitment success^26^. Oreti and Ripiro populations have tended to be more stable in recent decades^27^, while toheroa have now largely disappeared from the Kāpiti-Horowhenua coast^28^. This pattern of continued toheroa decline in Kāpiti-Horowhenua may account for the negative Fs and D values recorded for Waitarere, a population that may have limited larval connectivity with more northern toheroa.

Only two of the 20 haplotypes recorded in this study were found in more than one population and our analyses indicate statistically significant genetic differences between all regions. Genetic differences between North and South Island toheroa were the result of a complete lack of genetic diversity at Oreti in the South Island, a stark contrast to the relatively high levels of diversity recorded at each sampling location in the North Island (Fig. 3; Table 1). Divergences between northern and southern populations have previously been reported for New Zealand marine invertebrates^2^ including gastropods^29,30^, bivalves^10,31,32^, amphipod crustaceans^33^ and echinoderms^34^. However, divergence typically takes the form of regionally unique haplotypes or geographical differences in allele frequency. These divergences are often indicative of an independent evolutionary history and reflect the limited connectivity between northern and southern regions^35^. In contrast to these previous taxa, the single haplotype recorded for toheroa at Oreti in the South Island was the same haplotype most commonly recorded in the North Island. This is a genetic structure that has not previously been reported for any other New Zealand marine invertebrates, even those with similar reproductive modes and pelagic larval durations^2,23^. For example, an analysis of microsatellite loci for *P. subtriangulata*^10^, a surf clam that can co-occur with toheroa, revealed one largely undifferentiated population across its entire distribution (North Island and upper South Island). In contrast, population subdivision (including divergence between northern and southern populations) was evident in the genetic structure of estuarine *P. australis*^10^ (microsatellites) and *Austrovenus stutchburyi*^32^ (mtDNA COI) with multiple alleles recorded in all populations. For *A. stutchburyi*, a number of COI haplotypes were found throughout New Zealand, but at different frequencies in northern and southern populations, while other haplotypes were found exclusively in either the north or the south.

A lack of within-population genetic diversity, as we observed for Oreti toheroa, could result from isolation and inbreeding^36,37^ which is expected for the southern toheroa on account of their remoteness to North Island populations. If isolation had occurred in the distant evolutionary past we would expect some degree of divergence to have resulted after a period of isolation, and be manifested in the form of a set of unique genetic variants. Molecular clock estimates for marine molluscs suggest an evolutionary rate of between 2.3 and 4.6% per million years for the COI gene^38,39^. For the toheroa sequenced here, the six substitution steps between the central H1 haplotype and those at the farthest extent of the haplotype network could therefore represent somewhere between 270,000 and 539,000 years of toheroa evolution in northern New Zealand. In contrast, the lack of divergence or genetic diversity in southern toheroa suggests a far shorter evolutionary history and supports the notion of a more recent isolating event with no subsequent opportunity for divergence. Based on the above rates of evolution, divergence in Oreti toheroa from an ancestral population should be detectable after *c*. 45,000–91,000 years of isolation. Accordingly, the absence of unique haplotypes in the south suggests these populations were founded more recently.

Three scenarios could explain the geographic distribution of genetic diversity recorded in toheroa: First, that toheroa were formerly present along east or west coasts of the South Island (connecting northern and southern populations) and have disappeared from these areas due to factors such as over harvesting, the loss of suitable habitat or a changing climate. The occurrence of toheroa in Māori shell middens (domestic refuse heaps that indicate sites of human occupation or food processing activity) in the upper and central South Island would support this hypothesis if a range reduction had occurred after the settlement of New Zealand by Māori in approximately 1250 AD^40,41^. However, while toheroa shells are found both in middens and in natural deposits in the vicinity of present day toheroa populations (New Zealand Archaeological Site Recording Scheme), they have rarely been reported elsewhere. There is no archaeological evidence for toheroa populations at other locations and the habitat along east and west coasts of the South Island is, at present, largely unsuitable for toheroa (P. Ross *pers. obs*.).

A second possible explanation is that rare, long distance larval dispersal events from northern populations may have seeded toheroa in the south. While it is impossible to rule out this scenario, our understanding of coastal circulation patterns suggests that the delivery of toheroa larvae over distances of 800 km or more is unlikely^20,22^. However, if a founding dispersal event had occurred in the distant past (>45,000 to 91,000 years ago), genetic divergence from northern toheroa would be expected^42^. Alternatively, a more recent dispersal and founding event could have occurred, explaining the lack of divergence recorded in this study. However, there is currently no evidence to suggest that hydrodynamic conditions have changed such that long distance dispersal events would have only become possible in the recent past and this explanation seems unlikely.

A third possibility is that toheroa are not native to the South Island, but were translocated there by Māori. Early-Māori were prolific users of aquatic resources^43,44^ and were adept at food cultivation and translocation. The Polynesian explorers who settled New Zealand (the first Māori) brought with them plants (sweet potato, taro and yam)^45,46^ and animals (Pacific dogs and rats)^47^. Post settlement, Māori also domesticated and translocated numerous endemic plants^48^ and are thought to have translocated freshwater fish into lakes where they did not occur naturally^38^. Not surprisingly, it has been argued that translocation and active management of wild populations are part of Māori culture^49–51^. Despite the overwhelming evidence that cultivation and translocation were commonly used by early-Māori, the concept of pre-European translocation of marine species has received little attention and no attempts have been made to determine whether practices similar to those employed on land were also used in the marine estate – in this case, the cultivation of shellfish through translocation.

An examination of data sources typically outside of those utilised by practitioners of natural sciences (including legal and historical texts, popular media and interviews) has provided numerous accounts of toheroa translocation within both North and South Islands dating back at least as far as *c*. 1880 AD^52–55^. Based on this evidence, we now know that toheroa translocations took place. Translocations may even explain the small numbers of toheroa or their shells that have been reported at sites such as Pakiri or Karamea where toheroa are not found today (Fig. 1). However, at this time it is unknown whether the presence of toheroa in southern New Zealand, or on the east coast of the North Island, is solely a consequence of translocation. Further studies, incorporating archaeology, anthropology and molecular ecology (using more variable genetic markers), may address this uncertainty.

While the genetic structure of toheroa appears to be unique, there are few other relevant taxa for which similar data are available^2,23^. Population genetic analyses of New Zealand bivalves are limited to *A. stutchburyi*^32^, *Paphies australis*, *Paphies subtriangulata*^10^, and *Perna canaliculus* – a mussel with a population genetic structure that has to some extent been modified through modern aquaculture-related movements of broodstock and/or spat^33,56^. Consequently, we caution that other presently unstudied New Zealand bivalve taxa could have similar population genetic structures to that of toheroa and that these patterns may have arisen through natural biogeographic processes.

The reasons for the failure of toheroa to recover despite over 40 years of protection remain a mystery. The detection of a population genetic structure that is unique among New Zealand marine invertebrates^2^ adds to the intrigue surrounding this iconic species. While limited gene flow is just one of many factors potentially influencing toheroa population dynamics^14,57^, our study provides support for the hypothesis that South Island toheroa are genetically isolated. Furthermore, the limited sharing of haplotypes among northern populations may indicate limited connectivity at smaller spatial scales. As genetic diversity in northern toheroa is high, analyses that capture a greater proportion of this diversity will be required to provide estimates of larval exchange and will allow for comparisons with coastal and estuarine taxa. Such comparisons, and future population genetic studies, should incorporate thinking around the potential for humans to have influenced the distribution of marine species. If the translocation hypothesis is accepted, it could explain the disjointed modern distribution and population dynamics of toheroa and the limited success achieved in restoring what may, in some cases, be ecologically isolated populations located outside their natural distribution and preferred ecological niche.

## Methods

### Study sites and sample collection

Specimens for genetic analysis were obtained from the three main toheroa regions (Fig. 1). In northern New Zealand, toheroa were sourced from NMB (*n* = 10) and Ripiro Beach (*n* = 16), on the Kāpiti-Horowhenua coast from Waitarere Beach (n = 30) and in Murihiku from Oreti Beach (n = 31). Toheroa were collected during surveys commissioned by the Ministry for Primary Industries in 2009 (Oreti), 2010 (NMB) and 2011 (Ripiro) and under a customary harvest permit in 2015 (Waitarere). Toheroa were stored at −20 °C prior to DNA extraction.

### DNA extraction and sequencing

A 0.25–0.50 cm^2^ piece of adductor muscle was dissected from each specimen and genomic DNA extracted using the Zymo Research Genomic DNA II Kit (Zymo Research Corporation, Orange, CA, USA). The mitochondrial COI gene was amplified using the universal primers LCO1490 and HCO2198^58^. PCR amplifications were conducted in 10 µl reactions containing 4.8 µl Intron i-Taq 2x PCR master mix, 5 pmol of each primer and 1 µl of unquantified template DNA. PCR reactions consisted of an initial denaturing phase of 94 °C (4 min.), followed by 35 cycles consisting of 94 °C (60 s), 52 °C (90 s) and 72 °C (90 s) and a final extension period at 72 °C (5 min.). Unincorporated nucleotides and primers were removed by adding 2 units of Exonuclease I, 0.1 unit of Shrimp Alkaline Phosphatase and 2.7 µl H_2_O and incubating at 37 °C (30 min) then 80 °C (15 min). Sequencing reactions used Big Dye terminator sequencing chemistry (Applied Biosystems) on an Applied Biosystems 3130 Genetic Analyzer. DNA traces ends were generally of low quality and were trimmed and edited in Geneious Version 5.1.7 to produce a high quality alignment of 485 base pairs. Sequences have been deposited in the Barcode of Life Datasystems (BOLD) database under dataset DS-NZTOH (dx.doi.org/10.5883/DS-NZTOH) and cross-referenced to Genbank (accession numbers MH622204-MH622290). An additional 11 sequences from Oreti Beach (*n* = 5) and NMB (*n* = 6) were retrieved from the Barcode of Life Database (MOLNZ 183, 617–626).

### Population genetic analysis

Indices of genetic diversity were quantified using DnaSP Version 5^59^ and Arlequin Version 3.5.2.2^60^. For each population (Taitokerau (NMB and Ripiro), Waitarere and Oreti) we calculated the number of segregating sites (S), numbers of transitions (T_S_) and transversions (T_S_), the number of COI haplotypes (N_H_), the number of private COI haplotypes in a population (H_P_), haplotype diversity (H_D_), mean number of pairwise differences (k) and nucleotide diversity (π). A rarefaction analysis was generated in Analytic Rarefaction (Version 1.3; http://strata.uga.edu/software/anRareReadme.html; Holland 2003) to approximate the proportion of haplotype diversity captured by the current sampling regime. PopART Version 1.7 (http://popart.otago.ac.nz) was then used to generate a minimum spanning network to allow for visualisation of relationships between haplotypes and the geographic distribution of genetic diversity. Estimates of population pairwise F_ST_ values were then calculated in Arlequin to determine if any two populations differed significantly in their genetic composition. Tajima’s *D*^61^ and Fu’s Fs^62^ were calculated in DnaSP to test for deviation from the Wright–Fisher model of neutral evolution which can be indicative of either non-neutral evolution or population expansion or contraction under neutral evolution.

## References

[1] Cowen Robert K., Sponaugle Su (2009). Larval Dispersal and Marine Population Connectivity. Annual Review of Marine Science.

[2] Ross PM, Hogg ID, Pilditch CA, Lundquist CJ (2009). Phylogeography of New Zealand’s coastal benthos. N. Z. J. Mar. Freshw. Res..

[3] Selkoe KA, Toonen RJ (2011). Marine connectivity: A new look at pelagic larval duration and genetic metrics of dispersal. Mar. Ecol. Prog. Ser..

[4] Shanks AL (2009). Pelagic larval duration and dispersal distance revisited. Biol. Bull..

[5] Kingsford MJ (2002). Sensory environments, larval abilities and local self-recruitment. Bull. Mar. Sci..

[6] Shanks, A. L. Mechanisms of cross-shelf dispersal of larval invertebrates and fish in *Ecology of Marine Invertebrate Larvae* (ed L. McEdward) 323–367 (CRC Press, 1995).

[7] Shanks AL, Shearman RP (2009). Paradigm lost? Cross-shelf distributions of intertidal invertebrate larvae are unaffected by upwelling or downwelling. Mar. Ecol. Prog. Ser..

[8] Largier JL (2003). Considerations in estimating larval dispersal distances from oceanographic data. Ecol. Appl..

[9] Pinsky ML, Palumbi SR, Adrefouet S, Purkis SJ (2012). Open and closed seascapes: Where does habitat patchiness create populations with high fractions of self-recruitment?. Ecol. Appl..

[10] Hannan, D. A. *et al*. Genetic Connectivity Amongst New Zealand’s Open Sandy Shore and Estuarine Coastal Taxa. *New Zealand Aquatic Environment and Biodiversity Report* **172** (2016).

[11] Watts RJ, Johnson MS (2004). Estuaries, lagoons and enclosed embayments: Habitats that enhance population subdivision of inshore fishes. Mar. Freshw. Res..

[12] Levinton, J. S. Genetic divergence in estuaries in *Estuarine Perspectives* (ed V.S Kennedy) 509–520 (Academic Press, 1980).

[13] Ayre DJ, Minchinton TE, Perrin C (2009). Does life history predict past and current connectivity for rocky intertidal invertebrates across a marine biogeographic barrier?. Mol. Ecol..

[14] Ross PM (2018). The biology, ecology and history of toheroa (*Paphies ventricosa*): a review of scientific, local and customary knowledge. N. Z. J. Mar. Freshw. Res..

[15] Gadomski K, Moller H, Beentjes M, Lamare M (2015). Embryonic and larval development of the New Zealand bivalve *Paphies ventricosa* Gray, 1843 (Veneroida: Mesodesmatidae) at a range of temperatures. J. Molluscan Stud..

[16] Redfearn, P. Biology and Distribution of the Toheroa, *Paphies* (*Mesodesma*) *ventricosa* (Gray). *Fish. Res. Bull*. **11** (New Zealand Ministry of Agriculture and Fisheries, 1974).

[17] Cook, S. d. C. Class Bivalvia in New Zealand Coastal Marine Inverebrates Volume One (ed. Cook, S. d. C.) 471–541 (Canterbury University Press, 2010).

[18] Rapson AM (1952). The toheroa, *Amphidesma ventricosum* Gray (Eulamellibranchiata), development and growth. Aust. J. Mar. Fresh. Res..

[19] Rapson AM (1954). Feeding and control of toheroa (*Amphidesma ventricosum* Gray) (Eulamellibranchiata) populations in New Zealand. Aust. J. Mar. Fresh. Res..

[20] Chiswell SM, Rickard GJ (2011). Larval connectivity of harbours via ocean currents: A New Zealand study. Cont. Shelf Res..

[21] Heath RA (1985). A review of the physical oceanography of the seas around New Zealand. N. Z. J. Mar. Freshw. Res..

[22] Sutton PJH, Bowen MM (2011). Currents off the west coast of Northland, New Zealand. N. Z. J. Mar. Freshw. Res..

[23] Gardner, J. P. A. *et al*. Multi-species coastal marine connectivity: a literature review with recommendations for further research. *New Zealand Aquatic Environment and Biodiversity Report* **58** (2010).

[24] Berkenbusch, K., Abraham, E. & Neubauer, P. Distribution and abundance of toheroa in Southland, 2013–14. *New Zealand Fisheries Assessment Report* **2015/17** (2015).

[25] Williams, J., Ferguson, H. & Tuck, I. Distribution and abundance of toheroa (*Paphies ventricosa*) and tuatua (*P. subtriangulata*) at Ninety Mile Beach in 2010 and Dargaville Beach in 2011. *New Zealand Fisheries Assessment Report* **2013/39** (2013).

[26] McLachlan, A. & Brown, A. C. *The Ecology of Sandy Shores*. (Elsevier Inc., 2006).

[27] Williams, J., Sim-Smith, C. & Paterson, C. Review of factors affecting the abundance of toheroa (*Paphies ventricosa*). *New Zealand Aquatic Environment and Biodiversity Report* **114** (2013).

[28] Newcombe, E. *et al*. Kaimoana on beaches from Hōkio to Ōtaki, Horowhenua. *Manaaki Taha Moana Research Report* **22** *Cawthron Report* **2564** (2014).

[29] Veale AJ, Lavery SD (2011). Phylogeography of the snakeskin chiton *Sypharochiton pelliserpentis* (Mollusca: Polyplacophora) around New Zealand: are seasonal near-shore upwelling events a dynamic barrier to gene flow?. Biol. J. Linn. Soc..

[30] Goldstien SJ, Schiel DR, Gemmell NJ (2006). Comparative phylogeography of coastal limpets across a marine disjunction in New Zealand. Mol. Ecol..

[31] Apte S, Gardner JPA (2002). Population genetic subdivision in the New Zealand greenshell mussel (*Perna canaliculus*) inferred from single-strand conformation polymorphism analysis of mitochondrial DNA. Mol. Ecol..

[32] Ross PM, Hogg ID, Pilditch CA, Lundquist CJ, Wilkins RJ (2012). Population genetic structure of the New Zealand estuarine clam *Austrovenus stutchburyi* (Bivalvia: Veneridae) reveals population subdivision and partial congruence with biogeographic boundaries. Estuar. Coast..

[33] Knox MA, Hogg ID, Pilditch ID (2011). The role of vicariance and dispersal on New Zealand’s estuarine biodiversity: the case of Paracorophium (Crustacea: Amphipoda). Biol. J. Linnean. Soc..

[34] Waters JM, Roy MS (2004). Phylogeography of a highdispersal New Zealand sea-star: does upwelling block gene-flow?. Mol. Ecol..

[35] Wei K, Wood AR, Gardner JPA (2013). Population genetic variation in the New Zealand greenshell mussel: locus-dependent conflicting signals of weak structure and high gene flow balanced against pronounced structure and high self-recruitment. Mar. Biol..

[36] Avise, J. C. *Molecular Markers, Natural History and Evolution*. (Chapman & Hall, 1994).

[37] Keller LF, Waller DM (2002). Inbreeding effects in wild populations. Trends Ecol. Evol..

[38] Loeza-Quintana, T. Molecular clocks and rates of evolution in marine invertebrates *Unpublished PhD thesis* (University of Guelph, 2017).

[39] Crandall ED, Sbrocco EJ, DeBoer TS, Barber PH, Carpenter KE (2011). Expansion Dating: Calibrating Molecular Clocks in Marine Species from Expansions onto the Sunda Shelf Following the Last Glacial Maximum. Mol. Biol. Evol..

[40] Jacomb C (2014). High-precision dating and ancient DNA profiling of moa (Aves: Dinornithiformes) eggshell documents a complex feature at Wairau Bar and refines the chronology of New Zealand settlement by Polynesians. J. Arch. Sci..

[41] Wilmshurst JM, Anderson AJ, Higham TFG, Worthy TH (2008). Dating the late prehistoric dispersal of polynesians to New Zealand using the commensal Pacific rat. P. Natl. Acad. Sci. USA.

[42] Templeton AR (2008). The reality and importance of founder speciation in evolution. BioEssays.

[43] Leach, F. Fishing in pre-European New Zealand. *N. Z. J. Arch. Special Publication* (2006).

[44] McDowell, B. *Ikawai: freshwater fishes in Maori culture and economy* (Canterbury University Press, 2011).

[45] Horrocks M (2004). Polynesian plant subsistence in prehistoric New Zealand: a summary of the microfossil evidence. N. Z. J. Bot..

[46] Leach, H. *1,000 years of gardening in New Zealand*. (Reed, 1984).

[47] McKinnon, M., Bradley, B. & Kirkpatrick, R. *Bateman New Zealand historical atlas (Ko papatuanuku e takoto nei)* (David Bateman Limited, 1997).

[48] Shepherd Lara D., de Lange Peter J., Cox Simon, McLenachan Patricia A., Roskruge Nick R., Lockhart Peter J. (2016). Evidence of a Strong Domestication Bottleneck in the Recently Cultivated New Zealand Endemic Root Crop, Arthropodium cirratum (Asparagaceae). PLOS ONE.

[49] Dick J, Stephenson J, Kirikiri R, Moller H, Turner R (2013). Listening to tangata kaitiaki: the consequences of loss of abundance and biodiversity in Aotearoa, New Zealand. Mai Journal.

[50] Mccarthy A (2014). Local people see and care most? Severe depletion of inshore fisheries and its consequences for Māori communities in New Zealand. Aquat. Conserv..

[51] Moller, H. Customary use of indigenous wildlife – towards a bicultural approach to conserving New Zealand’s biodiversity in *Biodiversity*. (eds. McFagen B, Simpson P.) 89–125 (Department of Conservation, 1996).

[52] Habib, G. Part One. Report on Ngāi Tahu Fisheries Evidence. *Prepared for Waitangi Tribunal* (1989).

[53] New Zealand Herald. Toheroa experiment (13 August, 1934).

[54] New Zealand Herald. New toheroa bed (9 August,1926).

[55] Williams, J. E pākihi hakinga a kai: an examination of pre-contact resource management practise in Southern Te Wāi Pounamu. *Unpublished PhD thesis* (University of Otago, 2004).

[56] Apte S, Star B, Gardner JPA (2003). A comparison of genetic diversity between cultured and wild populations, and a test for genetic introgression in the New Zealand greenshell mussel *Perna canaliculus* (Gmelin 1791). Aquaculture..

[57] Ross PM, Pande A, Jones JB, Cope J, Flowers G (2018). First detection of gas bubble disease and Rickettsia-like organisms in *Paphies ventricosa*, a New Zealand surf clam. J. Fish Dis..

[58] Folmer O, Hoeh WR, Black MB, Vrijenhoek RC (1994). Conserved primers for PCR amplification of mitochondrial DNA from different invertebrate phyla. Mol. Mar. Biol. Biotech..

[59] Librado P, Rozas J (2009). DnaSPv5: a software for comprehensive analysis of DNA polymorphism data. Bioinformatics.

[60] Excoffier L, Lischer HEL (2010). Arlequin suite ver 3.5: a new series of programs to perform population genetics analyses under Linux and Windows. Mol. Ecol. Res..

[61] Tajima F (1989). Statistical method for testing the neutral mutation hypothesis by DNA polymorphism. Genetics.

[62] Fu YX (1997). Statistical tests of neutrality of mutations against population growth, hitchhiking and background selection. Genetics.

